# Surveillance and Management Strategies for African Swine Fever (ASF) in Central Luzon, Philippines

**DOI:** 10.3390/pathogens14100995

**Published:** 2025-10-02

**Authors:** Virginia M. Venturina, Romeo S. Gundran, Ronalie B. Rafael, Roderick T. Salvador, Marvin Bryan S. Salinas, Errol Jay Y. Balagan, Phebe M. Valdez, Alvin P. Soriano, Noraine P. Medina, Gemerlyn G. Garcia, Ma-Jian R. Dela Cruz, Lianne Kathleen P. Salazar, Lohreihlieh P. Parayao, Dante M. Fabros, Corrie C. Brown, Bonto Faburay

**Affiliations:** 1 Center for Transboundary Animal Diseases (CenTrAD), Central Luzon State University, Science City of Muñoz 3120, Nueva Ecija, Philippines; majrigos@yahoo.com (M.-J.R.D.C.); salazarlianne@gmail.com (L.K.P.S.); parayaolohreihlieh@gmail.com (L.P.P.);; 2 Department of Veterinary Paraclinical Sciences, College of Veterinary Science and Medicine, Central Luzon State University, Science City of Muñoz 3120, Nueva Ecija, Philippines; rsgundran@clsu.edu.ph (R.S.G.); rbrafael@clsu.edu.ph (R.B.R.); rtsalvador@clsu.edu.ph (R.T.S.); sorianoap@clsu.edu.ph (A.P.S.); gemerlyngarcia@clsu.edu.ph (G.G.G.); 3 Department of Basic Veterinary Sciences, College of Veterinary Science and Medicine, Central Luzon State University, Science City of Muñoz 3120, Nueva Ecija, Philippines; salinasmarvinbryan@clsu.edu.ph (M.B.S.S.); ebalagan@clsu.edu.ph (E.J.Y.B.); npmedina@clsu.edu.ph (N.P.M.); 4 Department of Veterinary Clinical Sciences, College of Veterinary Science and Medicine, Central Luzon State University, Science City of Muñoz 3120, Nueva Ecija, Philippines; valdezphebe@clsu.edu.ph; 5 LifeStock International, 550 Fortson Rd., Athens, GA 30606, USA; corrie@lifestock.org; 6 Foreign Animal Disease Diagnostic Laboratory, National Bio and Agro-Defense Facility, Animal and Plant Health Inspection Service, United States Department of Agriculture, Manhattan, KS 66505, USA; bonto.faburay@usda.gov

**Keywords:** African swine fever, diagnostics, Central Luzon Philippines

## Abstract

African swine fever (ASF) remains a major threat to swine production in Central Luzon, Philippines. This study assessed ASF detection and farm-level risk factors in Central Luzon using a risk-based surveillance framework. Pooled blood samples from five pigs per farm were collected in 277 farms across seven provinces and tested by real-time PCR. The analysis yielded an apparent farm-level prevalence of 26.7% (95% CI: 21.6–32.3), defined by one pooled 5-pig blood sample per farm. However, these values reflect risk-based surveillance outcomes rather than population-representative prevalence. Detection varied by province, with high rates in Bataan (80.5%) and Nueva Ecija (55.0%), moderate detection in Zambales (24.3%), lower detection in Pampanga (5.0%) and Tarlac (20.0%), and no positives in Aurora or Bulacan. Survey data were available for 201 farms. Firth-penalized logistic regression identified the absence of perimeter fencing as the only statistically significant predictor of ASFV detection. Veterinary oversight and consultancy showed protective but non-significant trends. These results highlight structural and professional biosecurity gaps, emphasizing the need for expanded veterinary outreach, fencing support, and training to mitigate ASF risk in smallholder-dominated production systems.

## 1. Introduction

The swine industry plays a vital role in the Philippine economy and food security, serving as a major source of animal protein and contributing significantly to the country’s livestock production. A considerable portion of this output is derived from smallholder farms, which dominate the national swine population structure [1]. However, this critical sector continues to face severe threats from infectious diseases, particularly African swine fever (ASF).

ASF is a highly contagious viral disease affecting domestic and wild pigs. While not transmissible to humans, its devastating impact on pig populations and the swine industry worldwide has been significant [2,3]. The causative agent is a large, enveloped, double-stranded DNA virus classified as the sole member of the genus *Asfivirus* under the family *Asfarviridae* [4]. Since its first identification in Africa in the early 1900s, ASF has evolved into a persistent transboundary threat, spreading across Africa, Europe, and Asia. In August 2018, ASF was reported in China, the world’s largest pork producer and consumer, marking its incursion into Asia [5,6]. The disease reached the Philippines in July 2019 and has since affected over 3800 barangays in 53 provinces by July 2022 [7].

Despite the known presence of ASF in the Philippines, critical knowledge gaps remain regarding its local epidemiology, particularly at the regional level. Understanding the disease’s distribution and the specific risk factors associated with its spread is essential for formulating effective and targeted control strategies. To address this gap, the current study aims to determine the prevalence of ASF and assess its associated risk factors among swine farms in Central Luzon. By integrating molecular diagnostics with basic mapping techniques, this work seeks to generate data that can support the design of region-specific interventions to mitigate ASF’s impact on Philippine swine farming.

## 2. Materials and Methods

### 2.1. Sample Size and Farm Selection

A cross-sectional study was conducted across the seven provinces of Central Luzon. Farms were recruited purposively using a risk-based strategy rather than random sampling, with inclusion based on accessibility, willingness to participate, and prior ASF exposure in the vicinity. This approach was necessary due to several practical constraints during the ASF epidemic. Movement restrictions, quarantine checkpoints, and strict farm-entry biosecurity measures limited access to many farms. Farmer willingness to participate was also variable, as those facing active or recent outbreaks were often reluctant to allow sampling. Although a province-level sample size of 37 farms was initially calculated under the assumption of simple random sampling (40% prevalence, 95% confidence, 6% precision), this calculation was used only to guide recruitment targets. Because the sampling was risk-based rather than random, the results are best interpreted as outcomes of targeted surveillance among the sampled farms, rather than as population-representative prevalence estimates.

### 2.2. Survey Questionnaire

A structured questionnaire was administered through in-person interviews at each participating site. The questionnaire collected data on farm geolocation, swine population, production type, and management or biosecurity practices. Each questionnaire response was linked to the corresponding biological samples through a unique identification system.

The questionnaire did not include items on wild suid interactions, as wild pigs are not present in most study provinces; thus, this factor was not considered relevant to the local epidemiological context.

### 2.3. Collection of Samples

Blood samples were collected from five pigs per farm. Selection of animals was based on previous ASF history within the farm or nearby surveillance zones. At least 5 mL of blood was obtained from each pig via the cranial vena cava. The five samples from each farm were pooled. Pooled blood samples were labeled using a standardized coding system (province, farm ID, and date) and stored in cool boxes during transport. Upon arrival at the laboratory, samples were stored at −80 °C until processing.

### 2.4. Nucleic Acid Extraction

DNA extraction was performed using TRIzol^®^ reagent (Invitrogen, Carlsbad, CA, USA) following the manufacturer’s protocol. All extractions were carried out in a sterile environment to avoid cross-contamination.

### 2.5. Real-Time PCR for the ASFV Detection

Detection of ASF virus DNA was conducted using the real-time PCR protocol by King et al. (2003) [8,9], targeting the ASFV p72 gene. The reaction mix per sample (25 µL total volume) included: 4.4 µL nuclease-free water, 12.5 µL 2× RT-PCR buffer, 0.425 µL each of forward and reverse primers (18 µM), 1.25 µL probe (5 µM), 1 µL 25X RT-PCR enzyme mix (AgPath-ID, Thermofisher, Austin, TX, USA), and 5 µL of DNA template. The inclusion of an RT step does not affect the assay performance [8]. Each PCR run included a positive extraction control (ASFV DNA, Cq = 27), a negative extraction control, and a no-template control. Amplifications were performed on a CFX96 Touch Real-Time PCR Detection System (Bio-Rad, Singapore, SG) under the following cycling conditions: 45 °C for 10 min, 95 °C for 10 min, followed by 45 cycles of 95 °C for 15 s and 60 °C for 45 s. Samples were considered ASFV-positive if they showed amplification curves similar to the positive control with a Cq value < 40. Table 1 shows the primers used for the molecular detection of ASF.

### 2.6. Statistical Analysis

All data—PCR results, farm profiles, and survey responses (Table S1)—were encoded in MS Excel and analyzed using R statistical software (version 4.2.3). Descriptive statistics were used to summarize farm characteristics and management practices. ASFV detection was expressed as the proportion of positive pooled samples among the sampled farms, with exact 95% confidence intervals reported as nominal values. Because sampling was risk-based rather than probabilistic, these detection rates may overestimate apparent prevalence, and the nominal 95% confidence intervals may not fully capture underlying uncertainty. They are presented for transparency but should be interpreted with caution. Given the purposive, risk-based sampling design (Section 2.1), these figures represent detection rates among the sampled farms and should not be interpreted as population-representative prevalence.

For the risk-factor analysis, only farms with both laboratory and completed survey data were included (201/277, 72.6%). Univariate associations between individual predictors and ASFV detection were first assessed using 2 × 2 cross-tabulations, crude odds ratios (ORs), and Fisher’s exact tests. A threshold of *p* < 0.10 was applied in the univariate analysis as a screening criterion to reduce the risk of excluding potentially important predictors, given the modest number of ASFV-positive farms (*n* = 52) and the possibility of type II errors. This liberal cutoff is consistent with common practice in veterinary epidemiology, where biologically plausible variables are carried forward to multivariable modeling. Final statistical significance in the multivariable Firth-penalized logistic regression was set at α = 0.05. Variables meeting the screening threshold, along with those identified a priori from the literature (e.g., absence of perimeter fencing, absence of a resident veterinarian, absence of a consultant), were considered for inclusion in the multivariable model.

To address sparse data in some provinces and reduce small-sample bias, multivariable models were fitted using Firth-penalized logistic regression. Collinearity was assessed using variance inflation factors (VIFs), all of which were <2, indicating no problematic overlap among predictors. Province was not included as a fixed effect in the regression due to sparse strata, but province-level heterogeneity is presented descriptively in the Results, with detection rates and exact 95% confidence intervals shown in Table 2. Adjusted ORs with 95% CIs and *p*-values are reported in Table 3.

To minimize the risk of false positives given the modest number of ASFV-positive farms (*n* = 74), the final multivariable model was restricted to a small set of biologically plausible predictors supported by prior literature. Other practices (e.g., entry of raw pork, insemination hygiene, potential mechanical transmission pathways) are reported descriptively but excluded from modeling due to lack of exposure timing and susceptibility to recall bias. Farm size and production type were considered as potential confounders but were not included in the final multivariable model due to sparse data and collinearity concerns, which risked model instability given the limited number of ASFV-positive farms.

A complete list of variables, coding schemes, and definitions is provided in Data S1 (Data Dictionary).

### 2.7. Spatial Mapping

Geographic locations of farms were recorded during field visits using handheld GPS units or smartphone GPS applications. Farm coordinates were linked to laboratory and survey data through unique farm identifiers. Spatial mapping and visualization were conducted in QGIS (version 3.34). Farms were symbolized as green (ASFV-negative) or red (ASFV-positive) circles, and surveillance coverage areas were shaded in blue. The final map included a scale bar, north arrow, and legend for clarity.

### 2.8. Ethical Considerations

Animal handling and sample collection were performed in compliance with Philippine animal welfare guidelines. Coordination with local veterinary offices ensured humane practices. No invasive procedures beyond standard veterinary protocols were used.

## 3. Results

### 3.1. ASFV Detection in Sampled Farms Across Central Luzon

This study analyzed pooled blood samples from swine farms across seven provinces in Central Luzon using a purposive, risk-based sampling strategy. Of the 277 pooled samples tested via real-time PCR, 74 were positive for ASFV (Data S2), corresponding to a detection rate of 26.7% among the sampled farms (95% CI: 21.6–32.3) (Table 2).

Detection rates varied across provinces. Among the sampled farms, ASFV was detected in 33 of 41 farms in Bataan (80.5%; 95% CI: 65.1–91.2) and in 22 of 40 farms in Nueva Ecija (55.0%; 95% CI: 38.5–70.7). Lower detection was observed in Zambales (24.3%; 9/37; 95% CI: 11.8–41.2), Tarlac (20.0%; 8/40; 95% CI: 9.1–35.7), and Pampanga (5.0%; 2/40; 95% CI: 0.6–16.9). No ASFV-positive farms were identified among those sampled in Aurora (0/40; 95% CI: 0–8.8) or Bulacan (0/39; 95% CI: 0–9.0).

**Figure 1:**
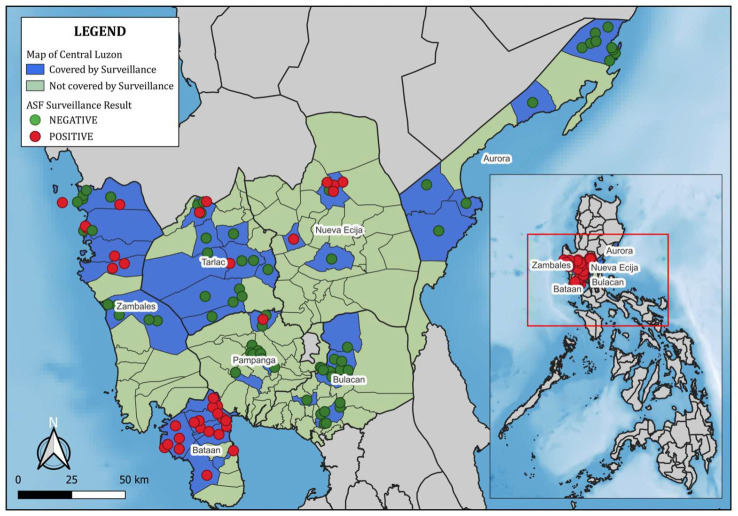
Geographic distribution of swine farms included in ASF surveillance across seven provinces of Central Luzon. Red circles = ASFV-positive farms; green circles = ASFV-negative farms. Blue shading indicates municipalities covered by surveillance. Mapping was performed in QGIS version 3.22.10.

Because farms were selected through a risk-based strategy, these figures represent surveillance outcomes among the sampled farms and should not be interpreted as population-representative prevalence. The absence of detected positives in Aurora and Bulacan should not be interpreted as evidence of freedom from ASF, but rather as reflecting the limits of the sampling design. Figure 1 shows the geographic distribution of sampled farms and ASFV detections across the region.

### 3.2. Farm Characteristics and Survey Coverage

Of the 277 farms tested for ASFV, 201 (72.6%) also completed the structured survey on demographics and biosecurity practices. These 201 farms were used for descriptive analysis and risk-factor modeling.

Farm production types were classified according to DA–BAI standards, with backyard farms defined as <20 total heads, semi-commercial farms as ≥20 but less than 50 heads, and commercial farms as >50 heads. Based on available survey responses, classification was possible for 186 farms: backyard (*n* = 97), semi-commercial (*n* = 52), and commercial (*n* = 37). The remaining farms could not be classified due to missing information on herd size.

Self-reported ASF awareness was high, with 181/194 respondents (93.3%) reporting knowledge of the disease. However, the consistent implementation of preventive practices was limited. Structural measures were incomplete: 61/191 farms (31.9%) lacked perimeter fencing, wheel dips were present on only 35/196 farms (17.9%), and footbaths were available on 73/194 farms (37.6%). Professional oversight was also constrained, with just 79/194 farms (40.7%) employing a resident veterinarian and 72/196 (36.7%) having a consultant.

Quarantine practices showed a bimodal distribution: 74/201 farms (36.8%) practiced short isolation of 1–7 days, while 41/201 farms (20.4%) used extended quarantine periods of more than 30 days (Figure S1). Risky behaviors remained common. Nearly half of farms practiced on-site slaughter (83/195, 42.6%), and 32/184 (17.4%) allowed pig traders to enter farm premises. Feeding of kitchen leftovers was reported in 36/193 farms (18.7%), and only 49/153 (32.0%) required health certificates when purchasing new animals. Vaccination programs for other swine diseases were reported by 106/194 farms (54.6%).

Experience in swine production also varied widely. A majority of respondents (125/201, 62.2%) reported 1–15 years of involvement, while only 16/201 farms (8.0%) had more than 40 years of experience (Figure S2).

### 3.3. Risk Factor Analysis

Univariate and multivariable analyses were restricted to the 186 farms with both ASFV laboratory results and completed survey responses (Table 3). Univariate analysis identified several biosecurity and professional oversight factors associated with ASFV detection (Table 3). Farms without a resident veterinarian (defined here as a veterinarian directly employed by the farm and regularly involved in herd management) (37/110, 33.6%) showed higher detection compared to those with one (15/76, 19.7%), with a crude OR of 2.06 (95% CI: 1.03–4.10). Similarly, farms without a consultant (defined as a visiting professional providing veterinary or management advice intermittently) (38/118, 32.2%) had higher detection than those with a consultant (14/68, 20.6%), with a crude OR of 1.83 (95% CI: 0.90–3.70). Variable definitions and coding are detailed in Data S1 (Data Dictionary).

In the multivariable model using Firth-penalized logistic regression (*n* = 186), the absence of perimeter fencing was the only factor that remained statistically significant (adjusted OR = 2.17, 95% CI: 1.13–4.39; *p* = 0.022). Farms without fencing had more than twice the odds of ASFV detection compared to those with perimeter fencing. The absence of a resident veterinarian (adjusted OR = 1.61, 95% CI: 0.73–3.70; *p* = 0.237) and the absence of a consultant (adjusted OR = 1.25, 95% CI: 0.55–2.87; *p* = 0.592) were associated with higher odds but did not reach statistical significance. Collinearity diagnostics indicated no problematic overlap among predictors (all VIF < 2).

Although only the absence of perimeter fencing remained statistically significant in the multivariable model, both the absence of a resident veterinarian and the absence of a consultant showed protective trends, while not reaching statistical significance. These findings suggest that professional oversight may still be epidemiologically relevant despite the limited statistical power of this dataset.

## 4. Discussion

This study provides important information on ASF detection patterns and farm-level risk factors in Central Luzon under a risk-based surveillance framework. Because farms were purposively recruited during the epidemic, the reported detection rates represent outcomes among sampled farms rather than population-level prevalence estimates. Such limitations are inherent to emergency field studies, where access, willingness to participate, and prior exposure risk determine which farms can be included. Even so, the findings remain valuable: they show provinces where ASFV was consistently detected, such as Bataan and Nueva Ecija; identify provinces with no positives despite targeted sampling, such as Aurora and Bulacan; and highlight farm-level practices—such as the absence of fencing, raw pork entry, and shared equipment—that were linked to the likelihood of detection. As these outcomes reflect risk-based surveillance rather than representative prevalence, comparisons across provinces should be regarded as descriptive patterns rather than inferential conclusions.

ASFV detection varied substantially across provinces. Bataan (80.5%) and Nueva Ecija (55.0%) had the highest detection rates, while Zambales (24.3%), Pampanga (5.0%), and Tarlac (20.0%) showed lower levels. Aurora (0/40) and Bulacan (0/39) recorded no ASFV-positive farms, but this should not be interpreted as evidence that the provinces were free of infection. Given the risk-based sampling strategy, the absence of positives more likely reflects sampling focus, farm participation, or reporting factors rather than true absence of disease. These results therefore provide a descriptive overview of where ASFV was or was not detected in the sample, rather than a definitive comparison of provincial prevalence.

ASF awareness among farmers was high, with more than 93% of respondents reporting knowledge of the disease. However, awareness did not consistently translate into practice, leaving many farms vulnerable. Fewer than half reported having veterinary oversight, and about one-third lacked perimeter fencing. Quarantine periods for newly introduced pigs were often shorter than the ASF incubation period, reducing their effectiveness for disease detection. Many producers were mid-career farmers with limited exposure to formal training in biosecurity, which may have contributed to inconsistent adoption of preventive measures. Risk behaviors such as on-site slaughter (42.6%) and trader access to farms (17.4%) were also common, creating additional opportunities for virus introduction and spread. Although wild suids are recognized as important reservoirs and vectors in other settings, they are not present in the study provinces and were therefore not included as a survey factor.

In the risk factor analysis, perimeter fencing was the only variable that consistently reduced the likelihood of ASFV detection, with farms lacking fences having more than twice the odds of being positive. This finding highlights the protective value of physical barriers, even under smallholder conditions. At the same time, studies in Europe have shown that fencing remains one of the weaker points in practice: Klein et al. (2024) reported that many farms had inadequate perimeter fences and poorly defined clean–dirty separation, reflecting financial and implementation challenges [10]. Although the presence of a resident veterinarian or a consultant showed protective trends in the univariate analysis, these associations did not remain statistically significant in the multivariable model. Several factors may explain this. First, the functional roles of resident veterinarians and consultants often overlap in practice, with some farms relying on visiting consultants for services similar to those provided by an employed veterinarian, making it difficult to separate their independent effects in a regression framework. Second, the modest number of ASFV-positive farms (*n* = 52 in the analytic dataset) limited the statistical power to detect moderate associations. Finally, professional oversight does not always ensure the consistent application of biosecurity recommendations, as reflected by the observed gap between high ASF awareness among farmers and variable implementation of preventive measures. Similar findings have been reported in other contexts, where veterinary services improved outcomes only when combined with strong structural and behavioral biosecurity measures [4,11,12]. Feeding-related practices, including swill feeding and the entry of raw pork, also remain biologically plausible risk factors, but they were not retained in the multivariable model because of uncertainties in exposure timing and the susceptibility of these variables to recall bias.

The policy implications are clear. ASF outbreaks cause severe losses for smallholders through herd depletion, restricted trade, and reduced household income. Strengthening farm resilience will require targeted measures that combine structural improvements with professional support. Evidence from other contexts shows that such investments are not only effective but also economically justifiable. Fasina et al. (2012) demonstrated that implementing full biosecurity in a smallholder system in Nigeria produced a benefit–cost ratio of about 29 in preventing ASF-related losses [11]. Similarly, Thao et al. (2022) reported that Vietnamese farms applying biosecurity practices achieved significant gains in economic efficiency [12], while Dione et al. (2017) showed that upgrading farm-level biosecurity in Uganda reduced ASF risk and supported market integration [13]. At the same time, experience from Europe highlights persistent weaknesses: it was found that many farms still lacked adequate perimeter fences and clear separation of clean and dirty areas, reflecting financial and implementation barriers [10]. These findings suggest that relatively low-cost measures such as subsidizing fencing materials, expanding veterinary outreach, and providing community-level training can provide measurable protection, provided that adoption is sustained and adapted to local conditions.

### Strengths and Limitations

A strength of this study is the integration of molecular diagnostics with detailed farm-level survey data collected during an active epidemic across seven provinces. This provided a unique opportunity to examine ASF detection patterns and biosecurity practices under real field conditions, offering insights that are directly relevant to smallholder production systems.

This study has several limitations that should be considered when interpreting the findings. First, farms were recruited through purposive, risk-based sampling rather than random selection. As a result, the reported detection rates reflect surveillance outcomes among the sampled farms and should not be interpreted as population-representative prevalence estimates. Second, only 201 of 277 farms completed the questionnaire, and further item non-response reduced the analytic sample to 186 farms for regression modeling. This may have introduced selection bias if non-responding farms differed systematically from those included. In particular, herd size information was incomplete for 15 farms, reducing the regression dataset from 201 to 186. When tested, herd size and production type were highly correlated with structural biosecurity variables (e.g., fencing, veterinary oversight), which led to unstable estimates given the modest number of ASFV-positive farms. For this reason, these variables were excluded from the final multivariable model, which may have limited our ability to fully adjust for farm-level confounding. Third, the modest number of ASFV-positive farms (*n* = 52) limited statistical power to detect moderate associations, particularly for overlapping roles such as resident veterinarians and consultants. Fourth, some survey variables—such as raw pork entry and insemination hygiene—lacked precise temporal linkage to sample collection and may be subject to recall or social desirability bias. Fifth, the cross-sectional design prevents causal inference, as exposures and outcomes were measured simultaneously. Another limitation is the case definition based on pooled sampling. ASFV detection at the farm level was determined from a single pooled blood sample of five pigs. While this approach was necessary for logistical and biosafety reasons during the epidemic, pooling can reduce effective assay sensitivity and specificity. Farms with very low within-herd prevalence could be misclassified as negative, whereas high-prevalence farms may be overrepresented. As such, the reported values represent apparent farm-level prevalence based on one pooled 5-pig blood sample, and binomial confidence intervals may underestimate uncertainty. Future studies should incorporate sensitivity analyses that vary within-farm prevalence and assay performance assumptions to better quantify the effect of pooling on apparent prevalence estimates. Finally, wild suids, which are important ASF vectors in other contexts, were absent from the study provinces and therefore not included in the survey instrument, limiting generalizability to settings where wild pig populations are present.

## 5. Conclusions

This study demonstrated substantial heterogeneity in ASFV detection across Central Luzon using a risk-based surveillance framework. Among the factors examined, perimeter fencing was the only statistically significant protective measure, while veterinary oversight and consultancy showed protective but non-significant trends, likely reflecting overlapping roles and variable adoption of recommended practices. The integration of laboratory testing with farm surveys revealed critical gaps in biosecurity, with many farms lacking both structural barriers and professional guidance.

These results highlight the need for targeted interventions, such as expanded veterinary outreach, support for fencing and other structural improvements, and farmer training in outbreak response. At the same time, the conclusions should be interpreted with caution given the study’s limitations, including purposive sampling, reliance on pooled blood samples for farm-level classification, the modest number of positive farms, and the cross-sectional design.

## Figures and Tables

**Table 1:** Oligonucleotide primers and fluorogenic probe for ASFV p72 gene.

Primer	Sequence (5′-3′)	Size (bp)
Forward	CTGCTCATGGTATCAATCTTATCGA	250
Reverse	GATACCACAAGATCAGGCCGT	
Probe	FAM-CCACGGGAGGAATACCAACCCAGTG-TAMRA	

**Table 2:** Detection of African swine fever virus (ASFV) in sampled swine farms by province, Central Luzon.

Province	Positive Farms (Cases)	Sampled Farms (Number of Pooled Samples)	Detection Rate (%)	95% CI	
				Lower Limit (%)	Upper Limit (%)
**Aurora**	0	40	0.00	0.00	8.81
**Bataan**	33	41	80.49	65.13	91.18
**Bulacan**	0	39	0.00	0.00	9.03
**Nueva Ecija**	22	40	55.00	38.49	70.74
**Pampanga**	2	40	5.00	0.61	16.92
**Tarlac**	8	40	20.00	9.05	35.65
**Zambales**	9	37	24.32	11.77	41.20

Cases = ASFV-positive farms, defined by pooled 5-pig blood samples tested by real-time PCR. Detection rates and exact 95% confidence intervals (CIs) are reported as nominal values. Farms were enrolled using a purposive, risk-based sampling strategy; results should not be interpreted as population-representative prevalence estimates. Risk-based sampling may inflate apparent prevalence, and the nominal 95% CIs do not account for the sampling design. Comparisons between provinces should be made with caution, as differences in sampling intensity and targeting may influence detection rates.

**Table 3:** Univariate and multivariable analysis of farm-level risk factors for ASFV detection in Central Luzon.

Variable	Category	ASF Positive	ASF Negative	Crude OR	95% CI (Crude) *	Adjusted OR (Firth)	95% CI (Adjusted)	*p*-Value (Adj)
Perimeter Fence	Absent	24	35	2.42	1.24–4.72	**2.17**	1.13–4.39	0.022
	Present	28	99					
Resident Veterinarian	Absent	37	73	2.06	1.03–4.10	1.61	0.73–3.70	0.237
	Present	15	61					
Consultant	Absent	38	80	1.83	0.90–3.70	1.25	0.55–2.87	0.592
	Present	14	54					

Cases = ASFV-positive farms; Crude odds ratios (ORs) with 95% confidence intervals (CIs) were estimated using Fisher’s exact tests. *p*-value (Adj) refers to the *p*-value obtained from the Firth-penalized logistic regression model including all listed predictors, which reduces small-sample bias and accounts for their simultaneous effects. * indicates statistical significance at *p* < 0.05, i.e., the 95% CI does not include 1.0. Adjusted ORs were obtained from a Firth-penalized logistic regression model including all listed predictors. Reference categories are “present” for each factor (Fence present, Veterinarian present, Consultant present). Analyses were restricted to farms with both laboratory and survey data (*n* = 186).

## Data Availability

The original contributions presented in this study are included in the article/Supplementary Material. Further inquiries can be directed to the corresponding author.

## Supplementary Materials

The following supporting information can be downloaded at: https://www.mdpi.com/article/10.3390/pathogens14100995/s1, Table S1: Profiles and management practices of swine farms in the Central Luzon region; Figure S1: Distribution of quarantine duration for newly introduced pigs among swine farms in Central Luzon; Figure S2: Distribution of years of experience in pig farming; Data S1: Data Dictionary (Codebook) detailing all variables, coding schemes, definitions, and reference categories used in the analysis; Data S2: Summary of qPCR results (Cq values) of ASFV detection in Region3.
