# Supplementary material for: Surveillance and Management Strategies for African Swine Fever (ASF) in Central Luzon, Philippines
Source: Pathogens. 2025 Oct 2;14(10):995. doi: 10.3390/pathogens14100995 (PMC12567132; doi:10.3390/pathogens14100995)
Supplement: Supplementary file 1 [file pathogens-14-00995-s001.zip › Figure S1 Distribution of quarantine duration for newly introduced pigs among swine farms in Central Luzon..pdf]

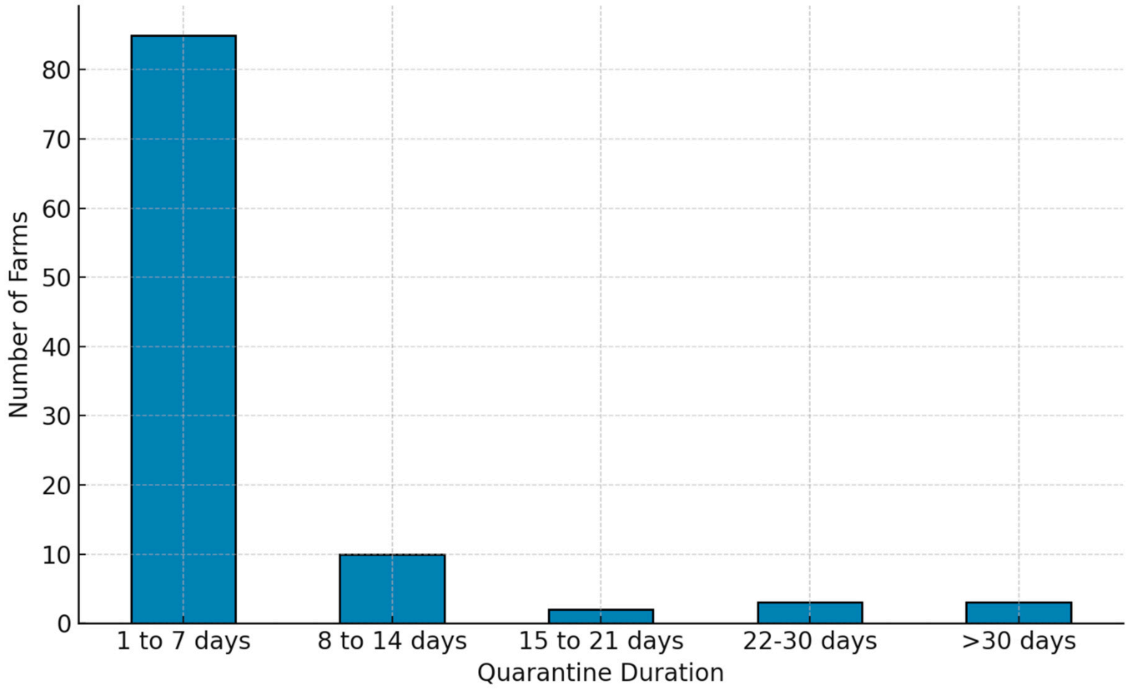

Figure S1. Distribution of quarantine duration for newly introduced pigs among swine farms in Central Luzon. The bar chart illustrates the number of farms implementing different quarantine durations for new stock. The majority of respondents reported implementing quarantine periods of 1–7 days (n = X) and more than 30 days (n = X), while fewer farms reported durations between 15–21 days. These findings highlight both short-term and extended quarantine practices, reflecting variability in biosecurity implementation across farms. The distribution suggests that while many farms recognize the need for isolation of new animals, there remains inconsistency in the duration of quarantine protocols.
