# Supplementary material for: Surveillance and Management Strategies for African Swine Fever (ASF) in Central Luzon, Philippines
Source: Pathogens. 2025 Oct 2;14(10):995. doi: 10.3390/pathogens14100995 (PMC12567132; doi:10.3390/pathogens14100995)
Supplement: Supplementary file 1 [file pathogens-14-00995-s001.zip › Figure S2 Distribution of quarantine duration for newly introduced pigs.pdf]

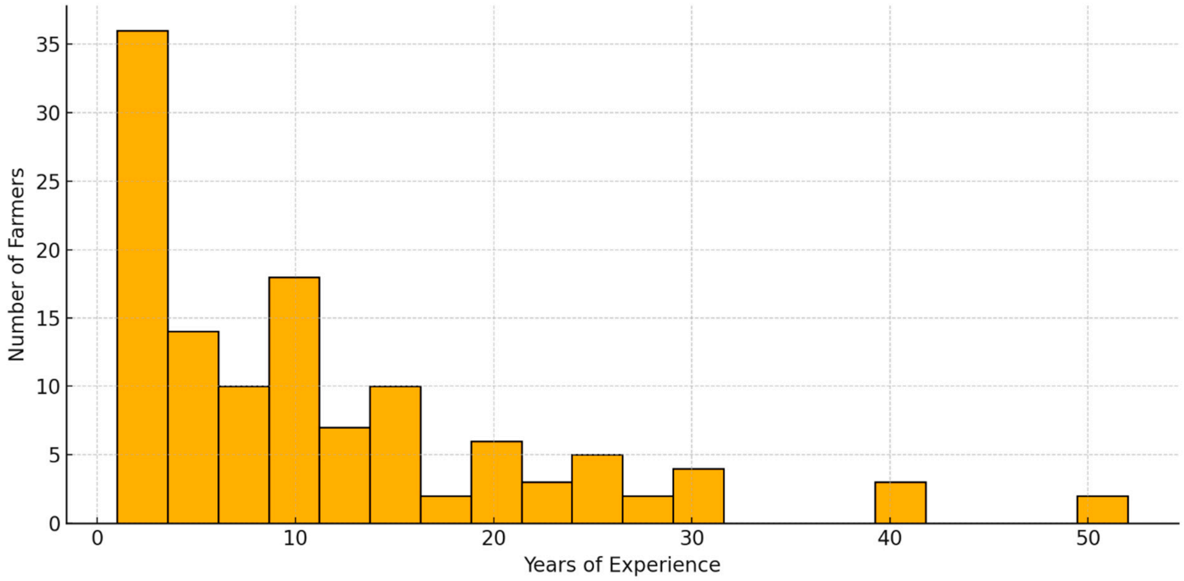

Figure S2. Distribution of years of experience in pig farming. The figure presents the frequency of self-reported experience durations in swine farming. The distribution is right-skewed, with most respondents indicating 1 to 15 years of experience. A smaller subset of farmers reported over 40 years of experience, highlighting variability in production tenure.
