# Supplementary material for: Surveillance and Management Strategies for African Swine Fever (ASF) in Central Luzon, Philippines
Source: Pathogens. 2025 Oct 2;14(10):995. doi: 10.3390/pathogens14100995 (PMC12567132; doi:10.3390/pathogens14100995)
Supplement: Supplementary file 1 [file pathogens-14-00995-s001.zip › Supplementary Data S1 Data Dictionary_Code Book.pdf]

Supplementary Data S1: Data Dictionary

Variable	Label	Type	Coding	Definition/Notes
ASFPositive	Farm-level ASFV detection (pooled rRT-PCR)	binary	1=yes, 0=no	Outcome; pooled from 5 pigs
Fence	Perimeter fence present	binary	1=present, 0=absent	Structural biosecurity; reference=present
Veterinarian	Resident veterinarian	binary	1=present, 0=absent	Directly employed vet; ref=present
Consultant	External consultant	binary	1=present, 0=absent	Visiting professional; ref=present
herdsize	Total headcount	integer	>=0, NA	Self-reported inventory
productiontype	Farm class	categorical	backyard<20; semi-commercial=20 - 49; commercial>=50	Derived from herdsize
ASFawareness	Heard of ASF	binary	1=yes, 0=no	Knowledge of ASF
wheeldip	Vehicle wheel dip/basin present	binary	1=yes, 0=no	Biosecurity measure
footbath	Footbath present	binary	1=yes, 0=no	At personnel entry
quarantinedaysgrp	Quarantine duration group	categorical		Grouped from survey
healthcertrequired	Health certificate required	binary	1=yes, 0=no	Incoming stock purchase
vaccinationprogram	Vaccination program present	binary	1=yes, 0=no	For other swine diseases
onsiteslaughter	Slaughter pigs on-site for sale	binary	1=yes, 0=no	High-risk practice
traderentry	Traders allowed to enter premises	binary	1=yes, 0=no	Biosecurity risk
kitchenleftovers	Feeding kitchen leftovers	binary	1=yes, 0=no	Swill feeding
toolsharing	Tools shared between buildings	binary	1=yes, 0=no	Biosecurity gap
watertesting	Water source tested	binary	1=yes, 0=no	Past 12 months
rawporkentry	Raw pork allowed to enter farm	binary	1=yes, 0=no	Buying pork outside the farm and consumed inside the piggery

AIpractice	Artificial insemination used	binary	1=yes, 0=no	Practice reported
AIsanitarydefined	AI sanitary protocol defined	binary	1=yes, 0=no	Only if captured in questionnaire
province	Province	categorical	Aurora, Bataan, Bulacan, Nueva Ecija, Pampanga, Tarlac, Zambales	Provincial location of the farm
sampledate	Sample date	date		Date of collection
farmid	Farm unique ID	string	Custom code	Links lab + survey data
