# Supplementary material for: Surveillance and Management Strategies for African Swine Fever (ASF) in Central Luzon, Philippines
Source: Pathogens. 2025 Oct 2;14(10):995. doi: 10.3390/pathogens14100995 (PMC12567132; doi:10.3390/pathogens14100995)
Supplement: Supplementary file 1 [file pathogens-14-00995-s001.zip › Supplementary Data S2 Summary of qPCR results (Cq values) of ASFV in Region3.pdf]

Supplementary data S2. Summary of qPCR results (Cq values) of African Swine Fever virus (ASFV) detection from different pig samples in Region III.

Table 1. Aurora Province

Sample No.	Cq	Result
A-B-24082023-0081	N/A	Negative
A-B-24082023-0082	N/A	Negative
A-B-24082023-0083	N/A	Negative
A-B-24082023-0084	N/A	Negative
A-B-24082023-0085	N/A	Negative
A-B-24082023-0086	N/A	Negative
A-B-24082023-0087	N/A	Negative
A-B-24082023-0088	N/A	Negative
A-B-24082023-0089	N/A	Negative
A-B-24082023-0090	N/A	Negative
A-B-24082023-0222	N/A	Negative
A-B-24112023-0147	N/A	Negative
A-B-24112023-0148	N/A	Negative
A-B-24112023-0149	N/A	Negative
A-B-24112023-0150	N/A	Negative
A-B-24112023-0151	N/A	Negative
A-B-24112023-0152	N/A	Negative
A-B-24112023-0153	N/A	Negative
A-B-24112023-0154	N/A	Negative
A-B-24112023-0155	N/A	Negative
A-B-24112023-0156	N/A	Negative
A-B-24112023-0157	N/A	Negative
A-B-24112023-0223	N/A	Negative
A-B-24112023-0224	N/A	Negative
A-B-24112023-0225	N/A	Negative
A-B-24112023-0226	N/A	Negative
A-B-24112023-0227	N/A	Negative
A-B-24112023-0228	N/A	Negative
A-B-24112023-0229	N/A	Negative
A-B-24112023-0230	N/A	Negative
A-B-24112023-0231	N/A	Negative
A-B-24112023-0232	N/A	Negative
A-B-24112023-0233	N/A	Negative
A-B-24112023-0234	N/A	Negative
A-B-24112023-0235	N/A	Negative
A-B-24112023-0237	N/A	Negative
A-B-24112023-0238	N/A	Negative
A-B-24112023-0239	N/A	Negative
A-B-24112023-0240	N/A	Negative

A-B-24112023-0241	N/A	Negative

Table 2. Bataan Province

Sample No.	Cq	Result
BT-B-0002	21.96	**Positive**
BT-B-0003	19.93	**Positive**
BT-B-0004	15.6	**Positive**
BT-B-0017	15.48	**Positive**
BT-B-0018	N/A	Negative
BT-B-0019	17.82	**Positive**
BT-B-0020	N/A	Negative
BT-B-0021	27.3	**Positive**
BT-B-0022	N/A	Negative
BT-B-0023	21.55	**Positive**
BT-B-0024	16.82	**Positive**
BT-B-0025	N/A	Negative
BT-B-0026	19.2	**Positive**
BT-B-0027	14.31	**Positive**
BT-B-0028	15.77	**Positive**
BT-B-0078	19.68	**Positive**
BT-B-0079	20.24	**Positive**
BT-B-0080	N/A	Negative
BT-B-0158	15.21	**Positive**
BT-B-0159	15.05	**Positive**
BT-B-0160	14.92	**Positive**
BT-B-0161	16.52	**Positive**
BT-B-0162	23.09	**Positive**
BT-B-0163	16.24	**Positive**
BT-B-0164	14.11	**Positive**
BT-B-0165	19.68	**Positive**
BT-B-0166	16.03	**Positive**
BT-B-0167	16.91	**Positive**
BT-B-0168	17.48	**Positive**
BT-B-0169	18.48	**Positive**
BT-B-0170	22.16	**Positive**
BT-B-0171	25.41	**Positive**
BT-B-0172	20.30	**Positive**
BT-B-0173	N/A	Negative
BT-B-0174	N/A	Negative
BT-B-0175	N/A	Negative
BT-B-0176	24.6	**Positive**
BT-B-0177	18.77	**Positive**
BT-B-0178	19.68	**Positive**

BT-B-0179	23.95	**Positive**

Table 3. Bulacan Province

BL-B-04072023-0012	N/A	Negative
BL-B-04072023-0013	N/A	Negative
BL-B- 04072023- 0014	N/A	Negative
BL-B- 04072023- 0015	N/A	Negative
BL- B-18012024- 0312	N/A	Negative
BL- B-18012024- 0313	N/A	Negative
BL- B-18012024- 0314	N/A	Negative
BL- B-18012024- 0315	N/A	Negative
BL- B-18012024- 0316	N/A	Negative
BL- B-18012024- 0317	N/A	Negative
BL- B-18012024- 0319	N/A	Negative
BL- B-18012024-0320	N/A	Negative
BL-B-24012024-0345	N/A	Negative
BL-B-24012024-0346	N/A	Negative
BL-B-24012024-0347	N/A	Negative
BL-B-24012024-0348	N/A	Negative
BL-B-24012024-0349	N/A	Negative
BL-B-24012024-0351	N/A	Negative
BL-B-17012024-0363	N/A	Negative
BL-B-25042024-0364	N/A	Negative
BL-B-25042024-0365	N/A	Negative
BL-B-25042024-0366	N/A	Negative
BL-B-25042024-0367	N/A	Negative
BL-B-25042024-0368	N/A	Negative
BL-B-25042024-0369	N/A	Negative
BL-B-25042024-0370	N/A	Negative
BL-B-25042024-0371	N/A	Negative
BL-B-25042024-0372	N/A	Negative
BL-B-25042024-0373	N/A	Negative
BL-B-25042024-0374	N/A	Negative
BL-B-25042024-0375	N/A	Negative
BL-B-25042024-0376	N/A	Negative
BL-B-25042024-0377	N/A	Negative
BL-B-25042024-0379	N/A	Negative
BL-B-25042024-0380	N/A	Negative
BL-B-25042024-0381	N/A	Negative
BL-B-25042024-0382	N/A	Negative
BL-B-25042024-0383	N/A	Negative
BL-B-25042024-0384	N/A	Negative

Table 4. Nueva Ecija Province

Sample No.	Cq	Result
NE-B-19062023-0005	N/A	Negative
NE-B-19062023-0006	N/A	Negative
NE-B-19062023-0007	37.31	**Positive**
NE-B-19062023-0008	N/A	Negative
NE-B-19062023-0009	36.49	**Positive**
NE-B-19062023-0010	N/A	Negative
NE-B-19062023-0011	36.22	**Positive**
NE-B-30092023-0096	25.20	**Positive**
NE-B-26102023-0218	24.44	**Positive**
NE-B-26102023-0219	24.99	**Positive**
NE-B-26102023-0220	29.31	**Positive**
NE-B-30112023-0242	N/A	Negative
NE-B-30112023-0243	21.12	**Positive**
NE-B-30112023-0244	N/A	Negative
NE-B-30112023-0245	N/A	Negative
NE-B-30112023-0246	N/A	Negative
NE-B-30112023-0247	36.45	**Positive**
NE-B-30112023-0248	19.27	**Positive**
NE-B-30112023-0249	N/A	Negative
NE-B-30112023-0250	20.40	**Positive**
NE-B-30112023-0251	40.44	**Positive**
NE-B-30112023-0252	35.44	**Positive**
NE-B-30112023-0253	21.90	**Positive**
NE-B-30112023-0254	25.20	**Positive**
NE-B-30112023-0255	N/A	Negative
NE-B-30112023-0256	21.01	**Positive**
NE-B-30112023-0257	16.56	**Positive**
NE-B-30112023-0258	17.21	**Positive**
NE-B-30112023-0259	27.49	**Positive**
NE-B-30112023-0260	35.45	**Positive**
NE-B-30112023-0261	14.82	**Positive**
NE-B-30112023-0262	N/A	Negative
NE-B-30112023-0263	N/A	Negative
NE-B-22112023-0277	20.36	**Positive**
NE-B-27062023-0119	N/A	Negative
NE-B-27062023-0120	N/A	Negative
NE-B-27062023-0121	N/A	Negative
NE-B-27062023-0122	N/A	Negative
NE-B-27062023-0123	N/A	Negative
NE-B-27062023-0125	N/A	Negative

Table 5. Pampanga Province

Sample No.	Cq	Result
P-B-19102023-0193	N/A	Negative
P-B-19102023-0194	N/A	Negative
P-B-19102023-0195	N/A	Negative
P-B-19102023-0196	N/A	Negative
P-B-20102023-0209	N/A	Negative
P-B-20102023-0210	N/A	Negative
P-B-26102023-0212	N/A	Negative
P-B-26102023-0213	N/A	Negative
P-B-26102023-0214	N/A	Negative
P-B-26102023-0215	N/A	Negative
P-B-26102023-0216	N/A	Negative
P-B-27102023-0217	N/A	Negative
P-B-22112023-0278	N/A	Negative
P-B-22112023-0279	N/A	Negative
P-B-22112023-0280	N/A	Negative
P-B-22112023-0281	N/A	Negative
P-B-22112023-0282	N/A	Negative
P-B-23112023-0283	N/A	Negative
P-B-23112023-0284	N/A	Negative
P-B-31012024-0325	N/A	Negative
P-B-31012024-0326	N/A	Negative
P-B-31012024-0327	N/A	Negative
P-B-31012024-0328	N/A	Negative
P-B-30012024-0329	N/A	Negative
P-B-30012024-0330	N/A	Negative
P-B-30012024-0331	N/A	Negative
P-B-30012024-0332	N/A	Negative
P-B-30012024-0333	N/A	Negative
P-B-30012024-0334	N/A	Negative
P-B-30012024-0335	N/A	Negative
P-B-30012024-0336	N/A	Negative
P-B-30012024-0337	N/A	Negative
P-B-30012024-0338	N/A	Negative
P-B-30012024-0339	N/A	Negative
P-B-30012024-0340	N/A	Negative
P-B-30012024-0341	N/A	Negative
P-B-30012024-0342	N/A	Negative
P-B-30012024-0343	N/A	Negative
P-B-21022023-0030	24.92	**Positive**
P-B-26072023-0062	20.78	**Positive**

Table 6. Tarlac Province

Sample No.	Cq	Result
T-B-13092023-0091	N/A	Negative
T-B-13092023-0092	N/A	Negative
T-B-13092023-0093	N/A	Negative
T-B-13092023-0094	N/A	Negative
T-B-13092023-0095	N/A	Negative
T-B-16092023-0182	33.55	**Positive**
T-B-06102023-0183	27.69	**Positive**
T-B-12102023-0184	36.12	**Positive**
T-B-12102023-0185	N/A	Negative
T-B-12102023-0186	N/A	Negative
T-B-16102023-0187	N/A	Negative
T-B-16102023-0188	26.55	**Positive**
T-B-16102023-0189	N/A	Negative
T-B-17102023-0190	24.43	**Positive**
T-B-17102023-0191	N/A	Negative
T-B-17102023-0192	23.19	**Positive**
T-B-24112023-0146	N/A	Negative
T-B-15012024-0292	N/A	Negative
T-B-15012024-0293	N/A	Negative
T-B-15012024-0294	N/A	Negative
T-B-24012024-0295	N/A	Negative
T-B-24012024-0298	N/A	Negative
T-B-24012024-0299	N/A	Negative
T-B-24012024-0300	N/A	Negative
T-B-24012024-0301	N/A	Negative
T-B-24012024-0302	N/A	Negative
T-B-24012024-0303	N/A	Negative
T-B-24012024-0305	N/A	Negative
T-B-24012024-0306	N/A	Negative
T-B-24012024-0307	N/A	Negative
T-B-24012024-0308	N/A	Negative
T-B-24012024-0309	N/A	Negative
T-B-24012024-0310	N/A	Negative
T-B-23012023-0029	16.92	**Positive**
T-B-17072023-0058	17.70	**Positive**
T-B-16012023-0097	N/A	Negative
T-B-18072023-0131	N/A	Negative
T-B-24072023-0132	N/A	Negative
T-B-27072023-0136	N/A	Negative
T-B-27072023-0137	N/A	Negative

Table 7. Zambales Province

Z-B-14012024-0221	N/A	Negative
Z-B-30112023-0264	N/A	Negative
Z-B-30112023-0265	36.56	**Positive**
Z-B-30112023-0266	21.01	**Positive**
Z-B-30112023-0267	N/A	Negative
Z-B-30112023-0268	N/A	Negative
Z-B-30112023-0269	N/A	Negative
Z-B-30112023-0270	N/A	Negative
Z-B-30112023-0271	N/A	Negative
Z-B-30112023-0272	N/A	Negative
Z-B-30112023-0273	N/A	Negative
Z-B-30112023-0274	N/A	Negative
Z-B-30112023-0275	N/A	Negative
Z-B-30112023-0276	N/A	Negative
Z-B-11112023-0285	N/A	Negative
Z-B-11112023-0286	N/A	Negative
Z-B-11112023-0287	N/A	Negative
Z-B-16012024-0321	N/A	Negative
Z-B-17012024-0322	N/A	Negative
Z-B-17012024-0323	N/A	Negative
Z-B-17012024-0324	N/A	Negative
Z-B-24012024-0353	N/A	Negative
Z-B-24012024-0354	N/A	Negative
Z-B-24012024-0355	N/A	Negative
Z-B-24012024-0356	N/A	Negative
Z-B-24012024-0357	N/A	Negative
Z-B-24012024-0358	N/A	Negative
Z-B-24012024-0359	N/A	Negative
Z-B-24012024-0360	N/A	Negative
Z-B-06092023-0037	18.30	**Positive**
Z-B-09062023-0038	23.49	**Positive**
Z-B-09062023-0039	19.70	**Positive**
Z-B-22062023-0042	26.28	**Positive**
Z-B-22062023-0047	19.83	**Positive**
Z-B-25072023-0060	25.50	**Positive**
Z-B-25072023-0061	18.58	**Positive**
Z-B-25072023-0134	N/A	Negative
