# Supplementary material for: Surveillance and Management Strategies for African Swine Fever (ASF) in Central Luzon, Philippines
Source: Pathogens. 2025 Oct 2;14(10):995. doi: 10.3390/pathogens14100995 (PMC12567132; doi:10.3390/pathogens14100995)
Supplement: Supplementary file 1 [file pathogens-14-00995-s001.zip › Supplementary Table S1 Profiles and management practices of swine farms in the Central Luzon region.pdf]

Supplementary Table S1. Profiles and management practices of swine farms in Central Luzon Region.

Profile/ Practices	Yes (%)	No (%)	Total
**Biosecurity Measures (External)**			
Has a complete perimeter fence	130(68.06)	61(31.93)	191
Prohibits entry of delivery trucks	151(84.35)	28(15.64)	179
Disinfects vehicles entering farm	70(39.32)	108(60.67)	178
Traders' trucks are empty	9(4.91)	174(95.08)	183
Allows visitors inside farm	46(24.33)	143(75.66)	189
Allows entry of other animals in pig pens	60(31.41)	131(68.58)	191
Borrows equipment from other farms	7(3.62)	186(96.37)	193
**Biosecurity Measures (Internal)**			
Uses disinfectants	161(82.98)	33(17.01)	194
Disinfect hands before entry to farm buildings	104(54.45)	87(45.54)	191
Have a farm wheel bath	35(17.85)	161(82.14)	196
Have footbaths	73(37.62)	121(62.37)	194
Wash and disinfect tools used for castration and vaccination	154(84.61)	28(15.38)	182
Has a designated boots for each farm building	84(44.44)	105(55.55)	189
Change of clothes before farm entry	100(52.35)	91(47.64)	191
**Farm Infrastructure & Management**			
Has a loading area for pigs sold	64(34.40)	122(65.59)	186
Has a farrowing pen	123(70.28)	52(29.71)	175
Has an isolation room for sick pigs	116(63.38)	67(36.61)	183
Visits healthy pigs first before attending to sick animals	42(68.85)	19(31.14)	61
Follows all in all out system	78(42.85)	104(57.14)	182
**Vector and Pest Control**			
Implements rat control program	50(26.04)	142(73.95)	192
Have breeding areas for mosquitoes	125(64.76)	68(35.23)	193
Mosquitoes in the farm	157(80.92)	37(19.07)	194
Have trees inside the farm	159(81.95)	35(18.04)	194
Have birds in the farm	137(70.25)	58(29.74)	195
**Breeding Practices**			
Purchase semen	67(34.89)	125(65.10)	192
Hires boar	73(37.43)	122(62.56)	195
Owns a boar for hire	26(13.33)	169(86.66)	195
Practice artificial insemination	70(35.89)	125(64.10)	195

**Feeding & Slaughter Practices**			
Feed pigs with kitchen left overs	36(18.65)	157(81.35)	193
Slaughter own pigs to sell in the market	83(42.56)	112 (57.44)	195
**Veterinary Health Management**			
Has a vaccination program	106(54.62)	88(45.36)	194
Vaccinate against HC	99(52.10)	91(47.89)	190
Tests farm water quality	31(15.97)	163(84.02)	194
Requires Health Certification when buying stocks	49(32.02)	104(67.97)	153
Has a Farm Consultant	72(36.73)	124(63.27)	196
Has a farm Veterinarian	79(40.72)	115(59.28)	194
**Disease Awareness**			
Aware of ASF	181(93.29)	13(6.70)	194
Aware of HC	141(72.68)	53(27.32)	194
Aware of Japanese B Encephalitis	43(22.27)	150(77.72)	193
**Visitor and Trade Protocols**			
Allows entry of sales representative in the farm	40(20.30)	157(79.70)	197
Allows entry of pork in the farm	33(17.18)	159(82.81)	192
Traders fetch pigs sold from the farms	95(53.07)	84(46.93)	179
allows entry of pig traders inside farm	32(17.39)	152(82.61)	184
Located close to other swine farms	76(56.29)	59(43.70)	135
